# Risk factors for toxocariasis during incarceration: the One Health intervention approach

**DOI:** 10.1038/s41598-023-45484-7

**Published:** 2023-11-09

**Authors:** Vamilton Alvares Santarém, Gabriel Luís Brucinski Pinto, Roberto Teixeira de Souza Filho, Isabella Braghin Ferreira, Susana Angélica Zevallos Lescano, William Henry Roldan Gonzáles, Jully Kosloski, Juliano Ribeiro, Rogério Giuffrida, Andrea Pires dos Santos, Louise Bach Kmetiuk, Alexander Welker Biondo

**Affiliations:** 1 Graduate College in Animal Sciences, University of Western São Paulo (UNOESTE), Presidente Prudente, São Paulo 19050-920 Brazil; 2 Graduate College of Cell and Molecular Biology, Department of Veterinary Medicine, Federal University of Paraná (UFPR), Curitiba, Paraná (PR) 80035-050 Brazil; 3 Institute of Tropical Medicine of São Paulo, University of São Paulo, São Paulo, 05403-000 Brazil; 4 Department of Comparative Pathobiology, Purdue University, West Lafayette, IN 47907 USA

**Keywords:** Immunology, Molecular biology, Diseases

## Abstract

Despite potential exposure to soil-transmitted helminths, especially when stray dogs and cats are present, toxocariasis in inmate populations remains to be established. Accordingly, the present study assessed the seroprevalence and associated risk factors of toxocariasis at the Women's State Penitentiary of Parana, Brazil. A total of 234/370 (63.2%; 95% CI 58.2–68.0) women inmates and 28/87 (32.2%; 95% CI 23.3–42.6) correctional officers were seropositive for anti*-Toxocara* spp. IgG by ELISA, with inmates 2.62-fold more likely positive (p = 0.00000026). The univariate model has identified that non-white (OR = 1.58, p = 0.047) and older than 39 years (OR = 1.28, p = 0.032) inmates were associated with mild but significant odds for seropositivity. Elementary or higher educational level was considered a protective factor for seropositivity. The presence of *Toxocara* spp. eggs was observed in 10/15 (66.7%) collected soil samples by centrifuge-flotation in Zinc Sulfate, and molecular analysis by PCR identified only *Toxocara cati* in these eggs. An intervention program was established with regular trap-neuter-release, with gradual removal for adoption (donation campaigns), treatment, and euthanasia when necessary (particularly due to advanced sporotrichosis). In addition, an educational awareness agenda was proposed, aiming to reduce soil contamination and accidental intake by the incarcerated population. A total of 40 feral cats were trapped, 20 males and 20 females, mostly adults. After trapping, 36 cats were neutered, treated, and microchipped in the Veterinary Teaching Hospital (VTH) at the Federal University of Paraná. Five trapped feral cats were euthanized, four diagnosed with advanced sporotrichosis, and one already neutered cat (not herein) with complications due to feline immunodeficiency virus (FIV). Female inmates presented higher seroprevalence for *Toxocara* spp. antibodies when compared to correctional officers, significantly associated with age, self-declared ethnicity (non-white), and lack of formal education. Despite the non-natural scenario of a state penitentiary, the One Health approach of *Toxocara* spp. has highlighted the interdisciplinary nature of the study and its relevance in understanding the complex interactions between human, animal, and environmental factors, particularly impacting female inmates. Further studies should establish the rate of inmate infection over time while deprived of liberty.

## Introduction

A disproportionate burden of infectious diseases, including HIV, tuberculosis, and hepatitis, have been reported among current and formerly incarcerated people worldwide^1^, particularly in low- and middle-income countries, primarily due to pre-existent conditions, confinement, and inadequate health access^2^. Not surprisingly, mortality by infectious diseases was five times higher among prison inmates than the general population in the State of Rio de Janeiro, Brazil^3^.

Persons deprived of liberty have also been among the most vulnerable to parasitic intestinal infections^4^. Helminths and intestinal protozoans may cause severe life-threatening diseases in inmates, particularly in low- and middle-income countries^5^. In Ethiopia, Africa, untrimmed hand fingernails in inmates were observed as a significant variable associated with infection by Ascaris lumbricoides, an important soil-transmitted helminth (S-THs)^6^. The prevalence of parasitic infections in the prison population of Ethiopia (88/121; 72.7%) indicated a consequence of poor environmental sanitation and lack of personal hygienic practices^7^.

Direct contact with soil, such as in agriculture activities (fruits and vegetable culture), may predispose S-THs infection in vegetable garden workers and schoolchildren^8^. Agricultural practices have also been increasingly used as skill practice and reintegration by prison labor programs during incarceration, aiming social reintegration^9,10^. In southern Ethiopia, a cross-sectional study has shown that inmates with no hand-washing practice after soil handling were twice more likely infected with intestinal parasites^5^.

Prison-based animal programs have been increasingly considered as another tool for stimulating sociability, responsibility, and patience among inmates^11,12^. For instance, the high-security Brisbane Women's Correctional Centre in Australia has adopted a program fostering cats to help one another towards a positive future^13^. As cats are the definitive hosts of Toxoplasma gondii and other zoonotic parasites, shedding parasitic structures (oocyst of protozoan and helminth eggs) via feces may contaminate soil and infect contacting persons^14^. Due to immunocompromising, toxoplasmosis has been 4 to fivefold higher in HIV-positive inmates in Java (OR = 4.3; 95% CI 1.112–16.204, p = 0.034) and in Malaysia (OR = 5.06; 95% CI 3.09–8.30)^15^.

Toxocariasis has been indicated among the most prevalent helminthic zoonosis worldwide, with an estimated 1.4 billion infected people, mostly living in subtropical and tropical regions^16^. A meta-analysis study has estimated in 19.0% the global seroprevalence for toxocariasis in the general population^17^. In Brazil, *Toxocara* spp. serosurvey has widely ranged even within age groups, from 4.2%^18^ to 63.6%^19^ in children, and from 8.7%^20^ to 71.8%^21^ in adults. In addition, toxocariasis has also varied in different Brazilian populations, with seropositivity found in 58/280 (20.7%) pregnant women^22^ and of 89/194 (45.9%) persons experiencing homelessness in southeastern^23^, and 212/328 (64.6%) persons living in traditional seashore areas^24^.

Transmission mainly occurs by ingestion of contaminated soil with *Toxocara* spp. embryonated eggs shed by dogs and/or cats, the definitive hosts^25^. Although human toxocariasis may be asymptomatic, injury by larval migration to major organs may cause clinical onset including liver and lungs (visceral toxocariasis), ophthalmic system (ocular toxocariasis) and central nervous system (neurotoxocariasis)^26^.

Despite the high potential exposure to S-THs, toxocariasis in the inmate population remains to be established. Accordingly, the present One Health study aimed to assess the seroprevalence of toxocariasis in people and animals, as well as the environmental risk factors for toxocariasis in incarcerated women in Brazil, and the influence of feral cats on the environmental contamination of in-prison areas by *Toxocara* spp. eggs.

## Results

### Characteristics of the study population

All inmates evaluated in the serosurvey were adults (range: 19–62 years old; median = 32), 26/370 (7.0%) were at the nursery, and 2/370 (0.5%) were pregnant at the time of the survey.

### Prevalence of anti-*Toxocara* IgG antibodies

Overall, 234/370 (63.2%; 95% CI 58.2–68.0) women inmates and 28/87 (32.2%; 95% CI 23.3–42.6) correctional officers were positive for *Toxocara* spp. antibodies, with the inmates being 2.62-fold more likely to be seropositive (p = 0.00000026; 95% CI 2.21–5.95) than correctional officers. At the nursery, 18/26 (69.2%) women inmates were seropositive, and both pregnant inmates were seronegative.

### Associated risk factors for *Toxocara* spp. infection

Although the univariate model identified that inmates aging from 27 to 39 years were less likely to be seropositive than the older than 39 years, this finding was not verified considering the younger inmates (< 27 years). Non-white inmates were more likely to have a seropositive test (OR = 1.58, 95% CI 1.03–2.43, P = 0.047) than white inmates (Table 1). According to univariable analysis, having an elementary or higher formal educational level was considered a protective factor for seropositivity in considering the inmates with the primary school as the reference.

**Table 1:** Associated risk factors for anti-*Toxocara* spp. (IgG) antibodies in inmates from a Women’s penitentiary in Southern Brazil (N = 370) by univariable and logistic regression statistical analysis.

Variables	Anti-*Toxocara* antibodies		Univariate analysis			Multivariate analysis	
	(Positive) n = 234	Negative n = 136	OR (95% CI)	P value	OR (95% CI)		P value
Age (years old)				0.032			
Older than 39	67 (28.6)	25 (18.4)	1.0 (ref.)		1.0 (ref.)		1.0 (ref.)
33 to 39	51 (21.8)	41 (30.1)	0.47 (0.25–0.86)		0.55 (0.28–1.10)		0.074
27 to 32	47 (20.1)	37 (27.2)	0.48 (0.25–0.89)		0.50 (0.25–0.97)		0.040
Younger than 27	69 (29.5)	33 (24.3)	0.78 (0.42–1.45)		0.84 (0.44–1.61)		0.604
Ethnicity				0.047			
White	116 (49.8)	83 (61.0)	1.0 (ref.)		1.0 (ref.)		1.0 (ref.)
Non-white	117 (50.2)	53 (39.0)	1.58 (1.03–2.43)		1.54 (0.98–2.44)		0.064
Education				< 0.001			
Primary	70 (29.9)	19 (14.0)	1.0 (ref.)		1.0 (ref.)		1.0 (ref.)
Elementary school	98 (41.9)	53 (39.0)	0.51 (0.27–0.92)		0.59 (0.31–1.10)		0.106
Graduate school	7 (2.99)	15 (11.0)	0.13 (0.04–0.36)		0.16 (0.05–0.47)		0.001
High school	59 (25.2)	49 (36.0)	0.33 (0.17–0.62)		0.41 (0.21–0.78)		0.008
Contact with cats in prison				0.170			
No	168 (75.7)	86 (68.3)	1.0 (ref.)				
Yes	54 (24.3)	40 (31.7)	0.69 (0.43–1.13)				
Wash hands before meal				0.164			
No	8 (3.45)	1 (0.76)	1.0 (ref.)		1.0 (ref.)		1.0 (ref.)
Yes	224 (96.6)	131 (99.2)	0.24 (0.01–1.36)		0.16 (0.01–0.99)		0.090
Ingestion of raw/undercooked meat				0.530			
No	138 (62.2)	84 (66.1)	1.0 (ref.)				
Yes	84 (37.8)	43 (33.9)	1.19 (0.75–1.89)				
Contact with soil in prison				0.605			
No	214 (93.4)	124 (95.4)	1.0 (ref.)				
Yes	15 (6.55)	6 (4.62)	1.43 (0.56–4.15)				
Biting nails				0.796			
No	144 (66.1)	82 (64.1)	1.0 (ref.)				
Yes	74 (33.9)	46 (35.9)	0.92 (0.58–1.45)				
Use of the outdoor sunlight yard				0.753			
No	50 (23.9)	27 (21.8)	1.0 (ref.)				
Yes	159 (76.1)	97 (78.2)	0.89 (0.52–1.50)				

1.0 (ref.): reference category; OR (95% CI): odds ratio (95% Confidence Interval).

**Figure 1:**
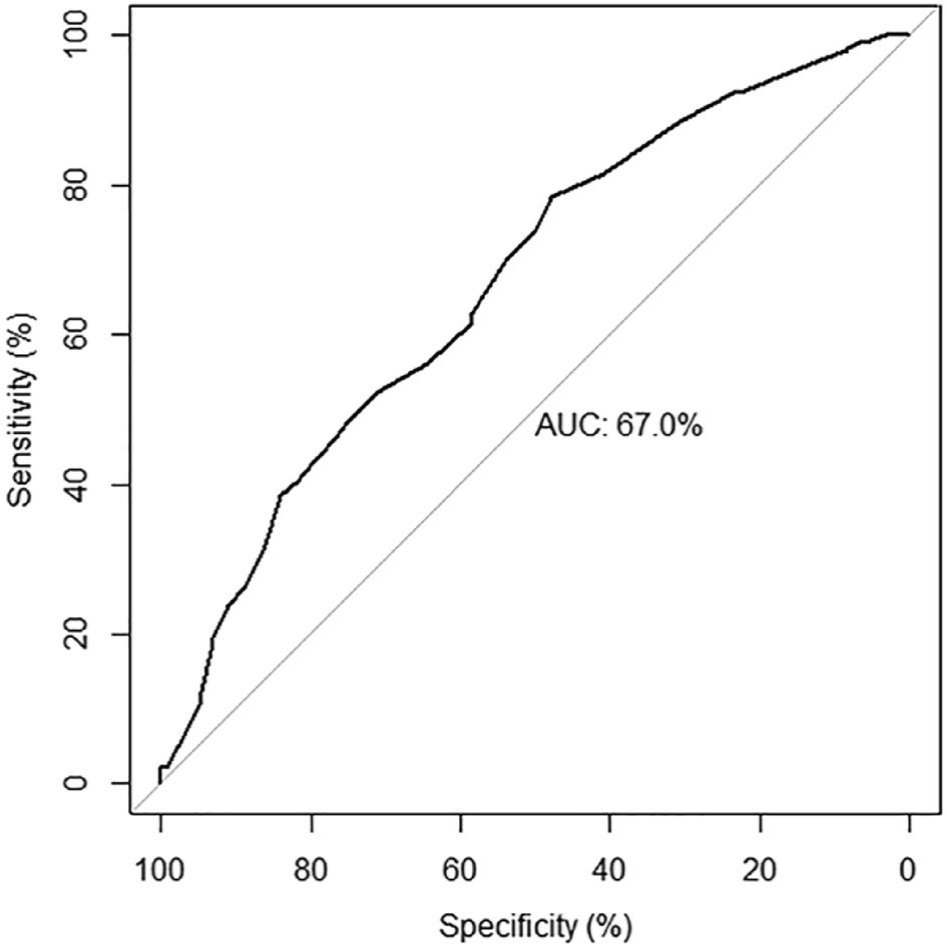
Receiver operating characteristic (ROC) curve assessing the accuracy of the multivariate logistic regression model for predicting seropositivity for anti-*Toxocara* spp. antibodies in a population of female inmates in a prison of Southern Brazil (area under curve (AUC): 0.67; 95% CI 0.612–0.727).

Logistic regression (multivariable analysis) identified educational level as an independent protective factor for seropositivity for anti-*Toxocara* spp. antibodies. The ROC results were presented and provided a comprehensive overview and accuracy of the findings (Fig. 1). However, the approach was of limited value for the outcome interpretation of logistic regression models, given that AUC has a probabilistic interpretation^27^.

None of the variables related to hygienic habits, practices, or having cats submitted to univariable analysis was associated with seropositivity in inmates. Regarding correctional officers, no risk factor, including socioeconomic, demographic characteristics, and habit variables, was significantly associated with seropositivity (Table 2).

**Table 2:** Associated factors for anti-*Toxocara* spp. (IgG) antibodies in correctional officers at a women’s penitentiary in Southern Brazil (N = 87) by univariate statistical analysis.

Variables	Anti-*Toxocara* antibodies		Univariate analysis	
	Positive (n = 28)	Negative (n = 59)	OR (95% CI)	P value
Ethnicity				0.493
White	11 (40.7)	18 (30.5)	1.0 (ref.)	
Non-white	16 (59.3)	41 (69.5)	0.64 (0.25–1.69)	
Education				1.0
Graduate or elementary school	21 (75.0)	45 (76.3)	1.0 (ref.)	
High School	7 (25.0)	14 (23.7)	1.08 (0.36–3.05)	
Use of the outdoor sunlight yard				0.261
No	10 (43.5)	20 (62.5)	1.0 (ref.)	
Yes	13 (56.5)	12 (37.5)	2.13 (0.71–6.60)	
Contact with cats in prison				0.636
No	10 (35.7)	22 (44.0)	1.0 (ref.)	
Yes	18 (64.3)	28 (56.0)	1.40 (0.54–3.77)	
Washing hands before meals				0.325
No	1 (3.70)	0 (0.00)	1.0 (ref.)	
Yes	26 (96.3)	56 (100)	. (.–.)	
Having meals served in the prison Restaurant				0.379
No	6 (21.4)	19 (33.3)	1.0 (ref.)	
Yes	22 (78.6)	38 (66.7)	1.80 (0.64–5.65)	
Drinking water at the prison				1.0
No	5 (17.9)	12 (20.3)	1.0 (ref.)	
Yes	23 (82.1)	47 (79.7)	1.16 (0.37–4.10)	
Ingesting raw/undercooked meat				0.280
No	20 (74.1)	35 (59.3)	1.0 (ref.)	
Yes	7 (25.9)	24 (40.7)	0.52 (0.18–1.39)	

1.0 (ref.): reference category.

The question regarding “drinking water at the prison” was applied only for correctional officers, as all inmates had the prison as the only drinking water source. Officers were offered additional options of “bring water from home”, “bottles of commercial water”, and “other”.

### *Toxocara* spp. eggs in soil samples

The presence of *Toxocara* spp. eggs was verified in 10/15 (66.7%) soil samples (Table 3).

**Table 3:** Abundance of *Toxocara* spp. eggs retrieved from soil samples collected in a women’s penitentiary in Southern Brazil.

Yard	Contaminated samples (%)	Number of retrieved eggs	Embryonated (%)
Between prison walls	2/5 (40.0)	17	2 (5.9)
Outdoor sunlight	5/5 (100)	651	284 (43.6)
Pregnancy and Nursery	3/5 (60.0)	22	3 (13.7)

A total of 690 eggs were recovered from soil samples, with an average of 1.7 eggs per gram of analyzed soil, with soil from the outdoor sunlight yard showing a higher level of contamination and number of embryonated eggs. Genetic amplification of the eggs collected revealed DNA only of *T. cati* in all three selected areas.

### Feral cats

No feces samples were collected from feral cats for assessment of fecal parasites. Feral cat handling by volunteers was considered of high zoonotic risk, particularly due to no history of previous rabies vaccination of feral cats, no guaranteed rabies protective titters of volunteers, and a cat sporotrichosis epidemic at the time.

During the study, 40 feral cats were trapped, 20 males and 20 females; within these cats, one kitten was 5–6 months old, nine cats were young adults, and 30 were adult cats. After trapping, 16 cats were neutered, treated, microchipped, and promptly released back during the study; 20 intact cats were sent for treatment and neutering at the Veterinary Teaching Hospital (VTH) of the Federal University of Paraná. A total of four/40 (10.0%) trapped feral cats were euthanized, including two intact and two neutered diagnosed with advanced sporotrichosis. In addition, one already neutered (not performed herein) female cat was severely sick even with treatment and was euthanized after diagnosed with feline immunodeficiency virus (FIV).

## Discussion

The present study has assessed a highly vulnerable population in the first comprehensive study of *Toxocara* spp. in inmates worldwide, with a relatively high overall seroprevalence (263/370; 63.2%). These results suggest a high exposure of participants to toxocariasis, with 2.62 times more likely to be seropositive than correctional officers, demonstrating a significant difference in toxocariasis exposure.

Recent serosurveys in other vulnerable adult groups of the same area in Southern Brazil have also shown high prevalence with 212/328 (64.6%) seropositive persons in populations of islands and mainland cities^24^, and with 247/344 (71.8%) in a rural population of Rio Grande do Sul State^21^. On the other hand, a lower prevalence (28/194; 14.4%) was observed in adults living in other rural communities of southeast Brazil^28^. Although with differences in sample size, comparisons can be made among these studies as all applied similar ELISA protocols to detect anti-*Toxocara* IgG, minimizing interstudy differences^24,28^. Geographical location, lifestyle, and age population may also be accounted for these differences in seroprevalence^21^.

Toxocariasis has been primarily transmitted by ingestion of soil contaminated with *Toxocara* spp. eggs^29^. In urban environments, *T. cati* eggs have been more commonly recovered from soil samples^30^ as observed in parks of Portugal^31^, Northwestern^32^ and Southwestern Iran^33^. *T. cati* eggs buried along with the feces by cats can protect eggs from desiccation and adverse weather conditions, prolonging the longevity and increasing the timeframe for human transmission^30^. A recent study of *T. cati* infection (377/846; 44.6%) in free-roaming cats of Oklahoma, United States, highlighted the cat's role in environmental contamination by parasite shedding^34^.

Expectedly, cat contact has been an important risk factor for toxocariasis^35–37^. As in the study herein, around 70 free-roaming feral cats circulated daily in outdoor and indoor areas, including all yards, hallways, and corridors. An "island-effect" has been hypothesized for human *Toxocara* spp. infection in seashore Brazilian islands, proposed to describe an exacerbated pathogen transmission due to island daily exposure and confinement, particularly favored by sand-inhaling transmission and tropical climate^24^. Although an island-effect may explain the exacerbated *Toxocara* spp. transmission herein by intense daily exposure and confinement as result of incarceration, further studies should be performed to fully establish such effect, particularly assessing over time incarceration and in-prison work as associated risk factors for infection in inmates and correctional officers, respectively. Analysis of soil samples from different in-prison yards revealed a high level of environmental contamination with *Toxocara* spp., mainly in the outdoor sunlight yard, where 256/333 (76.9%) inmates referred to having access. The majority of retrieved eggs were embryonated and plausibly considered viable and infective. PCR analysis revealed the presence only of *T. cati* eggs. Surprisingly, variables including contact with cats in prison, contact with soil, and visiting the outdoor sunlight yard were not associated with seropositivity. Despite such findings, the high number of cats and embryonated eggs observed in soil samples along with handling activities have drawn a favoring scenario for toxocariasis transmission to inmates and corroborated with the high seroprevalence observed herein^38^.

The univariate analysis in the present study has pointed out that inmates aged from 27 to 39 years were less likely to be seropositive than the older than the reference group (39 years). Studies have shown that seropositivity odds for toxocariasis may increase with aging due to exposure over time in the adult population^21,39^. Although the ELISA test used herein detected anti-*Toxocara* antibodies (IgG) with no clear distinction between recent and past infection^40^, a recent study with Brazilian pregnant women has shown that antibody avidity of persistent antibodies may indicate a past infection^22^.

In the present study, a higher former educational level was a significant protective factor for *Toxocara* spp. infection, corroborating with previous studies showing that education and socioeconomic status were inversely associated with seroprevalence^22,39,41–43^. In addition, non-white inmates had increased odds of being seropositive compared with white inmates (OR = 1.58, 95% CI 1.03–2.43, P = 0.047). In a serosurvey carried out in northern China, with four ethnic groups, individuals of Korean ethnicity showed the highest seroprevalence (20.58%), followed by Mongol (18.89%), Han (14.21%) and Manchu (11.22%)^44^. Maternal ethnicity was also a risk factor for seropositivity (non-Afro vs. Afro-Ecuadorian: OR 0.65, 95% CI 0.47–0.91, P = 0.011) of children in Ecuador^45^. Studies carried out in the United States of America with adults^41,43^, and children^46^ have also shown the influence of ethnicity in seropositivity. Unfortunately, the Brazilian women’s inmate population has been mostly self-declared as Black or Brown, with a poor and low level of formal education^47^, which altogether makes toxocariasis a high-risk zoonosis in such a vulnerable population.

Associated risk factors such as onychophagia^48^ and ingesting raw meat ^2535^ have been associated with toxocariasis. Moreover, no hand washing practice after handling soil^5^ and untrimmed hand fingernails^6^ were also observed as variables significantly associated with S-THs in Ethiopian inmates. However, no association of risk factors was verified herein for not washing hands before meals, as inmates mostly referred to it (355/364; 97.5%), nor for the habit of biting nails, as a slight majority used to do it (226/346; 65.3%), nor for raw or undercooked meat, since meat offered to inmates at the prison was adequately cooked. Nonetheless, parasitic infections in prison populations worldwide have been considered a reflection of poor environmental sanitation and personal hygienic practices^7^. As hygiene conditions and manners at the women’s penitentiary were considered the best in the Piraquara Complex, the situation may differ in male units, which accounted for most complex incarceration with 6,300/6,670 (94.4%) inmates and 691/778 (88.8%) correctional officers (director, personal information). The individual ethnicity herein was obtained by self-declaration, as previously accessed with other vulnerable populations by our research group, including islanders^24^, homelessness^23^, indigenous^49^, and with hoarding behavior^50^.

Incarcerated pregnant women have long posed a significant challenge to perinatal and nursing health^51^. Human congenital transmission of *Toxocara* spp. was restricted to two ocular toxocariasis cases, including a neonate presenting retinopathy^52^ and a five-week-old infant with strabismus^53^. Nevertheless, both vertical transmission of *T. canis*^54,55^ and intrauterine passage of *T. cati* larvae^56^ were experimentally demonstrated in murine models. Moreover, maternal *Toxocara* spp. infection during pregnancy has been suggested to cause miscarriage in pregnant women^57^. Although *Toxocara* spp. seroprevalence among pregnant women in Brazil has ranged from 6.4%^58^ to 20.7%^22^, the present study had only two auto-declared pregnant inmates, both serologically negative. However, 27/370 (7.3%) were at the nursery, with babies up to six months of age. Thus, further (probably multicentric) studies should be conducted to establish the prevalence fully, associated risk factors, and clinical onset of toxocariasis in incarcerated pregnant and nursery women.

As testing limitations, the study herein used *T. canis* excretory-secretory (TES) antigen for the ELISA test, which has not differentiated *T. canis* and *T. cati* infections due to their high homology degree of surface and excretory-secretory antigens. Molecular approach (PCR) revealed *T. cati* in genetic material of the eggs retrieved from soil of the three investigated areas in prison unit, which may have overcome such limitations. No dogs were found inside the prison, supporting these findings. In addition, ELISA does not provide differentiation between acute and latent infection of *Toxocara* spp.^40^.

As overall prison limitations, database of aerial maps, imprisonment locations, and global positioning systems (GPS) of cells and sectors were considered highly sensitive and unavailable due to internal ethical and security issues. As inmate data limitations, database of time of first reclusion, time served, and reincarcerations were also considered sensitive and unavailable, which has impaired assessment of such variables as associated risk factors for toxocariasis. Refusal, inaccuracy, or falseness of questionnaire responses by inmates and correctional officers may have partially led to biased results, such as self-declared ethnicity and access to raw or undercooked meat. Additionally, it is worth considering whether some inmates have opportunities to leave the facility for specific reasons, potentially exposing them to external sources of infection.

As another limitation, the studied prison was the only woman penitentiary of seven units and accounted for only 370/6,670 (5.6%) inmates and 87/778 (11.2%) correctional officers, not representative of the Piraquara Complex as a whole. As (another) limitation, the differences between the analysed variables were very small and perhaps do not allow concluding the risk factors that allow a profile of women to be more susceptible than another, except in relation to the level of schooling, which has proven to be a protective factor for infections.

As animal limitations, no feces samples were collected from feral cats to assess fecal parasites. As mentioned, feral cat handling by volunteers was considered of high zoonotic risk, mainly due to no previous rabies vaccination of feral cats, no guaranteed rabies protective titters of volunteers, and a cat sporotrichosis epidemic at the time. As the level of soil contamination and *T. cati* egg abundance in soil samples at different in-prison yards insured presence of infected feral cats and was considered more important for inmate and correctional officer exposure, researchers decided not to include feces samplings as a safety measure to prevent unnecessary bites, scratches, and direct contact with feral cats.

No feces samples were directly collected from feral cats to assess fecal parasites, as a safety measure to prevent unnecessary contact, handling, scratching, and biting from feral cats. Considered of high zoonotic risk, feral cats herein had no previous records of rabies vaccination, volunteers had no guaranteed rabies protective titers, and cases of cat sporotrichosis were confirmed in the penitentiary at the time. In addition, cat feces were not collected from the ground either, as cat habits of bury their feces made difficult to access individual feces and differentiate from soil samples, and the impossibility to determine whether feces and frequency of *Toxocara* spp. infection were from all or just a few cats. Nonetheless, the level of soil contamination and *T. cati* egg abundance in the tested soil samples has indirectly shown the presence of contaminated feces and infected feral cats in different in-prison yards, confirming inmate and correctional officer exposure. Thus, researchers decided not to include feces samplings collected directly from the cats as a safety measure to prevent unnecessary bites, scratches, and direct contact with feral cats. Herein, no attempt was done to collect feces from the ground due to the habit of cats bury their feces, making it difficult the access of the fecal material, and the impossibility to precise the frequency of *T*. *cati* infection.

The integrated approach based on the One Health concept was a significant contribution of the present study. Similarly, a recent One Health approach study applied to leptospirosis in a prison of South Africa has detected *Leptospira interrogans* in two inmates, along with positive rodent and water samples, confirming the within-prison cycle transmission^59^. Thus, the One Health approach may become mandatory to fully understand the occurrence of zoonotic pathogens and the multifaceted connections among human, animal, and environmental elements, even in an artificial setting such as a women’s state penitentiary.

In conclusion, our results have shown a high seroprevalence for toxocariasis in women inmates. The findings of our study further indicated that interventions based on the One Health approach, such as removing feral cats in prisons, may contribute to mitigating the soil contamination by *T. cati* eggs and, consequently, the risks of toxocariasis during incarceration.

## Methods

### Ethics statement

The authors confirm that the procedures comply with national and Brazilian regulations. The present study was approved by the Ethics Committee of the Federal University of Paraná for Research with human beings, according to the terms of Resolution nº 196/96 of the National Health Council, on July 28, 2020 (CAAE: 31,676,020.3.0000.0102, protocol number: 4.177. 728). In addition, feral cat capture, transportation, treatment, neutering, and return were approved by the Ethics Committee of Animal Use (protocol number 022/2022) of the Federal University of Parana. Reporting of results follows the recommendations of the ARRIVE guidelines. The investigation was carried out in coordination with the Penitentiary Department of the Piraquara Complex, which officially included the study as part of official activities and the Federal University of Paraná. Before participating in the present study, each human gave informed written consent for using the results in research.

As the study herein assumed veterinary intervention as counterpart and ethical research, the Shelter Veterinary Medicine service and the Veterinary Teaching Hospital at the Federal University of Paraná assumed full free-of-charge assistance to feral cats at the Penitentiary from April 16th 2020 to May 6th 2022. Feral cats were weekly trapped, microchipped, examined, treated (when necessary), vaccinated, dewormed, given pour-on anti-flea, neutered, and released back in the prison when healthy, in compliance with the animal welfare recommendations made by the District Attorney, Paraná State Public Ministry (Environment) at the time. Kittens and docile cats were sent for adoption.

### Study population and area

A total of 370 women deprived of liberty and 87 correctional officers were included in the study. The human blood sampling of women deprived of liberty and correctional officers was carried out in four consecutive days to safely accommodate inmate movement and officer shifts, from October 19th to 22nd 2020, at the Women’s State Penitentiary of Parana State, a major Brazilian complex with 6,670 men and women inmates supervised by 778 correctional officers, accommodated in seven different units at the time. The Complex is located in the Piraquara municipality (25°24′59" S, 49°04′46" W), part of the metropolitan area of Curitiba, the capital of Paraná State and the eighth largest city in Brazil with around 3,2 million habitants.

### Blood sample collection and epidemiological information

The participants were sampled after signing a consent form and completing an epidemiological questionnaire. Blood samples (10 mL from each participant) were then collected by cephalic venipuncture, placed in a tube with serum separator gel, centrifuged at 800 g for 5 min, and subsequently, serum separated and kept at -20º C until processing. Blood samples were drawn by certified nurses in partnership with the Piraquara City Secretary of Health.

The epidemiological evaluation of the inmates and correctional officers was based on a questionnaire designed to assess the toxocariasis risk factors (Supplementary material 1; Table 4).

**Table 4:** Content for assessing the potential exposure to toxocariasis.

Topics	Gathered information
Socioeconomic and demographic characteristics	Age, educational level, ethnicity
Domestic animals	Cat contact in prison
Hygienic habits	Washing hands before meals
Practices	Contact with soil; ingestion of raw meat; biting nails; frequenting solarium of prison

For data collection, participants were informed about the study and were informed about the confidentiality of their identities and the right to refuse participation at any time. The participants formalized the authorization by signing the Free and Informed Consent Term in compliance with Resolution No. 441/2012 of the National Brazilian Health Council.

As the study herein assumed veterinary intervention as counterpart and ethical research, the Shelter Veterinary Medicine service and the Veterinary Teaching Hospital at the Federal University of Paraná assumed full free-of-charge assistance to feral cats at the Penitentiary from April 16, 2020, to May 6, 2022. Feral cats were weekly trapped, microchipped, examined, treated (when necessary), vaccinated, dewormed, given pour-on anti-flea, neutered, and released back in the prison when healthy, in compliance with the animal welfare recommendations made by the District Attorney, Paraná State Public Ministry (Environment) at the time. Kittens and docile cats were sent for adoption.

### Laboratory testing of samples

#### Obtaining *Toxocara* antigen for ELISA

Roundworms (*T. canis*) were obtained from naturally infected puppies that spontaneously shed adult forms. Parasites were exposed to 1% sodium hypochlorite (5 min.) to remove debris and washed with 0.9% saline (3 min.). After washing, the anterior third of the worm was dissected to collect eggs. In vitro production of *T. canis* excretory-secretory (TES) antigen^60^ and protein concentration^61^followed protocols previously described.

In order to mitigate cross-reactivity with other ascarids, serum samples were preincubated with *Ascaris suum* adult worm extract (AWE)^60^. *A. suum* (adult forms) were macerated and one part 1.5 M NaOH added to nine parts of water, making a final concentration of 0.15 M. Following a 2 h incubation at room temperature, pH of the material was neutralized with 6 M HCI and centrifuged (18.500 g; 20 min at 4 °C). After lipidic remotion under ether treatment, the supernatant was filtered in 0.22 µm membranes (Millipore Co., Burlington, MA, USA).

### Serological testing

All serum samples were pre-incubated for 30 min at 37 °C with an AWE solution (25.0 µg/µL) in 0.01 M phosphate-buffered saline (PBS, pH 7.2) containing 0.05% Tween 20 (PBS-T) (Sigma, St. Louis, MO, USA), to avoid cross-reaction with Ascaris spp. antigen. Polystyrene 96-well microtiter plates (Corning, Costar, New York, USA) were coated for 1 h at 37 °C and then for 18 h at 4 °C, with 2.0 µg/ul per 100 uL/well of TES antigens in 0.06 M carbonate-bicarbonate buffer (23,24), at pH 9.6 and subsequently blocked for 1 h at 37 °C with 3% Molico® skimmed milk PBS-Tween 5%. After adsorption with A. suum somatic antigen, anti-Human IgG (Fc-specific) peroxidase antibody produced in goat (Sigma A6029) was added at a 1:5000 dilution (45 min. at 37 °C), performing three 5-min washes.

The reaction was detected using the substrate o-phenylenediamine (0.4 mg/mL, Sigma). The reaction was stopped by adding 2N sulphuric acid. Absorbance was read at 492 nm. A serum previously shown to be non-reactive (negative control) and a known reactive serum (positive control) were tested in each plate. Antibody levels were expressed as reactivity indexes (RI), which were calculated as the ratio between the absorbance values of each sample and the cut-off value.

### Collecting and retrieving *Toxocara* spp. eggs in soil samples

Soil samples were collected considering the presence of soil in the set points, in three different areas belonging to the woman's unit: (1) the outdoor sunlight-concreted yard, fully accessed by inmates; (2) the playground in front of the pregnancy and nursery building, accessed by inmates from late pregnancy up to six months after-born babies; and (3) the yard between prison-walls, not accessed by inmates. At the sunlight-concreted yard, soil samples were composed by accumulated soil and sand, mostly collected between concrete plaques. A minimal distance of three, five, and eight meters was adopted for collecting the samples, respectively, in the areas 1, 2 and 3, considering the dimensions of each area. Although divided by area in hierarchic groups, feral cats had fully in-prison access in most areas and outside access through bars of the underground sewer system and the front gate walls.

In each set, five soil samples of approximately 100 g were randomly collected at a depth of 5–10 cm, transferred to a plastic bag, and stored under refrigeration (4 °C) until analysis. Analysis of soil samples was performed following the protocol previously established^31^, with some modifications. Briefly, 20 g of each soil sample was weighed, rinsed with anionic detergent (100 mL of 5% Tween-80), homogenized, and allowed to stand overnight. Next, the contents were filtered in metallic sieves (300, 212, 90, and 38 μm) and washed in running water (10 min). The material retained in the sieve of 38 μm was collected and transferred to a graduated tube, filled with 10 mL of distilled water, and centrifuged (873 × g for 5 min). The supernatant was discarded, and the pellet was suspended and subjected to a centrifuge-flotation technique by mixing with 12 mL of zinc sulfate solution (d = 1.35 g/cm^3^). After a five-minutes-flotation-period, 5 mL of the supernatant was carefully transferred to two other graduated tubes (2.5 mL each) with the addition of 10 mL of distilled water and another centrifugation (873 × g for 5 min). The last aforementioned washing process was repeated three times to remove the sulfate solution.

The soil pellet was then evaluated under optical microscopy (10× and 40× objective) for counting and morphological evaluation of the eggs. *Toxocara* spp. eggs were classified according to the stage of development: viable (intact egg with content), non-viable (egg not intact or with a damaged wall), embryonating (egg with two or more cell divisions), and embryonated (egg containing larvae of the parasite).

All the *Toxocara* spp. Eggs observed under the microscope were collected using a micropipette (50 μL), transferred to a microtube (2 mL), and kept at − 20 °C until DNA extraction for PCR.

### DNA extraction and PCR assay

Genomic DNA from isolated *Toxocara* spp. eggs recovered from soil was extracted using a commercial purification kit (PureLinkTM Microbiome DNA, Invitrogen, Waltham, MA, USA) following the manufacturer's instructions, with modifications. Briefly, eggs were transferred to a microtube containing lysis buffer solution, homogenized (Scilogex D160 Homogenizer, Bedfordshire, England), and incubated in 20 μL proteinase K solution (65 °C for 16 h) for eggs disruption. Positive controls comprised female *T. canis* and *T. cati* genomic DNA extracted from eggs from naturally infected dogs and cats.

DNA concentration was determined through 260/280 nm absorbance measures using a commercial spectrophotometer (NanoDrop 2000, Thermo Scientific, Waltham, MA, USA).

Ribosomal DNA (rDNA) region (partial sequences of ITS1 and ITS2) was subjected to molecular analysis using the following previously described primers^62^: *T. canis*: (Forward: 5′-CTC GAG TCG ACG AAG TAT GTA C-3′; Reverse: 5′-AAT TGG GCC GCC CAT CAT AC-3′), and *T. cati* (Forward: 5′-GTA AGA TCG TGG CAC GCG TAC GTA-3′; Reverse: 5′-TCT TTG ATG TCA AGA CTT CAG CGC-3′).

Amplification reactions were performed in 25 μl reaction mixtures containing 0.02 mM of deoxynucleotide, 10 µm of each primer, 30 mM MgCl2, 1 U of Taq DNA polymerase (Invitrogen), 2 µL 10 × PCR Buffer, and approximately 50 ng of DNA template. PCR was performed using a PCR-programmed thermal cycler (Multigene, Labnet International, Edison, NJ, USA). Amplifications were performed under the following conditions: initial cycle at 94 °C for 60 s, followed by 35 cycles of denaturation at 94 °C for 60 s, annealing at 55 °C for 45 s, extension by polymerase at 72 °C for 30 s, and a final cycle at 72 °C for 5 min.

The gel electrophoresis was performed by adding 25 μl of the PCR products with loading buffer (5×) along with 100 bp DNA ladder to a 1.5% ethidium bromide-stained agarose gel for 90 min at 63 V.

### Statistical analysis

All the statistical analyses were performed using blorr package in R v.4.2.3, as previously established^63^. Toxocariasis seropositivity was compared between women deprived of liberty and correctional officers using Pearson's chi-square test. To access risk factors related to seropositivity in inmates, outcome data were initially categorized (variables shown in Table 1) and submitted to the univariate analysis (Pearson Chi-Squared Test or Fisher's exact test). Variables presenting statistical significance lower than 0.20 in the univariate model were included in logistical regression (multivariate analyses) for assessing the contribution of the risk/protective factors studied to the likelihood of *Toxocara* spp. seropositivity. To improve the final model, the predictor variables were tested for collinearity and the presence of influential values. Variables with a high degree of collinearity were excluded from the logistic model (inflation factor of variance higher than 4.0). From the regression coefficients for each predictor variable, odds ratio values were estimated per point and a 95% confidence interval. The best-fitting model was considered the one that included significantly associated variables (p-value < 0.05) and minimized the Akaike Information Criterion (AIC) value. A significant level of 5% was adopted for all statistical tests. The ROC curve presenting the model's accuracy is shown in Fig. 1.

### Supplementary Information

Supplementary Information.

## Data Availability

The datasets generated during and/or analyzed during the current study are available from the corresponding author on reasonable request.
