# Supplementary material for: Risk factors for toxocariasis during incarceration: the One Health intervention approach
Source: Sci Rep. 2023 Nov 9;13:19470. doi: 10.1038/s41598-023-45484-7 (PMC10636119; doi:10.1038/s41598-023-45484-7)
Supplement: Supplementary file 1 — Supplementary Information. [file 41598_2023_45484_MOESM1_ESM.pdf]

Supplementary material 1. Epidemiological questionnaire for correctional officers (A) and inmates (B) to assess the toxocariasis risk factors.

A. Correctional officers: Interviewer: _______________ Date: ____/____/_______ Sample identification: ____

**1. Name:**	**2. CPF:**	
**3. City of Origin:**	**4. Function.**	
**5. Date of birth:**	**6. Gender:** ( ) Female ( ) Male ( ) Other:	
**7. Marital Status:** ( ) Single ( ) Married ( ) Widowed ( ) Stable Union ( ) Separated		
**8. Ethnic data:** ( ) White ( ) Black ( ) Brown ( ) Indian ( ) Other		
**9. Education:**		
( ) No literacy	( ) Incomplete elementary school	( ) Complete high school
( ) Incomplete primary school	( ) Elementary complete	( ) Incomplete higher
( ) Complete primary school	( ) Incomplete high school	( ) Graduated
**10. Number of people in the house:**	**11. Minimum wage income:**	
**12. Do you have direct contact with prey?** ( ) Yes No.	**13. Weekly frequency:**	**14. Number of prey per week:**
**15. Do you have any chronic illness (Comorbidities):**		
**16. Address:**		
**17. During your activities, do you go to the solarium?** ( ) Yes. **Weekly frequency:** ( ) No		
**18. Do you have contact with the cats in the penitentiary?** ( ) Yes. **Week frequency?** ( ) No		
**19. Do you wash your hands before meals?** ( ) Yes. **How?** ( ) No		
**20. Do you have animals at home:** ( ) Yes. **Which?** ( ) No		
**21. Do you eat prison meals?** ( ) Yes No. **Weekly frequency:**		
**22. Do you consume penitentiary water?** ( ) Yes No.		
**23. Do you wash fruits and vegetables before consumption?** ( ) Yes. **How?** ( ) No		
**24. Do you eat raw or rare meat?** ( ) Yes. ( ) No. **Species:**		
**25. Are you pregnant?** ( ) Yes. Time: ( ) No	**26. Have you had children?** ( ) Yes, how many? ( ) No	
**27. Have you ever had a miscarriage?** ( )Yes Times: No ( ). **Cause?**		
**28. Were you working at PFP when you got pregnant?** ( ) Yes No		
**29. Did you attend the penitentiary day care?** ( ) Yes. **Time in months:** ( ) No		
**30. Do you feel pain in your eyes?** ( ) Yes No	**31. Any other vision problems?** ( )No.( )Yes. Which?	
**32. Fever for more than 2 weeks without clinical diagnosis?** ( ) Yes: ( ) Continuous ( ) Recurrent ( ) No		
**33. Have you ever been bitten by muquirana (body lice)?** ( ) No. ( ) Yes. If so, in the penitentiary? ( ) Yes No		
**34. Do you see rats in the penitentiary?** ( ) No ( ) Yes. Local:	**34. Ever been bitten by rats?** ( ) Yes No	
**35. Have you had contact with someone suspected/positive for COVID-19?** ( ) Yes. **Date:** ( ) No		
**36. Have you been tested for COVID-19 (SARS-CoV-2)?** ( ) Yes. **When?** ( ) No		
**37.If yes, which test?** ( ) Quick test (blood). **Result:**	( ) PCR (swab). **Result:**	
**38. Do you use a face mask?** ( ) Yes No	**39. Temperature:**	
**40. Have you had any of the clinical signs below in the last 3 months?**		
( ) Fever	( ) Headache	( ) Conjunctivitis
( ) Dry cough	( ) Diarrhea	( ) Breathing difficulty
( ) Fatigue	( ) Abdominal pain	( ) Loss of smell/taste
( ) Chest pain	( ) Body/joint pain	( ) Loss of speech or movement
**Symptoms onset date:**	**Do you still have any other symptoms?** ( ) Yes. Which? ( ) No	
**Fill with system data:**		
**Have you been tested for COVID-19 (SARS-CoV-2)?** ( ) Yes. Date? ( ) No		
**If yes, which test?** ( ) Quick test (blood). **Result:**	( ) PCR (swab). Lacen ( ) UFPR ( ). **Result: CT:**	
**Type of contract and length of time working at PFP:**		
**Have you worked in other units? ( ) Yes. Time: Which** ? ( ) No		
**Comorbidities:**		
**Comments:**		

TERMS OF FREE AND INFORMED CONSENT - Agents

We Prof. Alexander Welker Biondo (researcher in charge), Gabriel Luís Brucinski Pinto (Doctoral student) and from the Federal University of Paraná responsible for the research, as well as project participant Louise Bach Kmetiuk (Doctor), Juliano Ribeiro (Doctor), we are inviting you to participate in the studies entitled "Prevalence of anti- *Toxoplasma gondii* antibodies in a prison population and potential risk factors" and "Molecular and serological monitoring of SARS-CoV-2 in prisoners, prison guards and contacting cats of the Women's Penitentiary of Paraná".

a) The objective of this research is to evaluate the exposure of inmates, penitentiary agents and cats in contact with the Female Penitentiary of Paraná to SARS-CoV-2 through molecular detection. For this purpose, samples will be collected with swabs from the oropharynx/nasopharynx of detainees, employees and cats to verify the presence of the infectious agent. As well as to evaluate the prevalence of anti-T *oxoplasma gondii* antibodies in the occupants of the Piraquara Women's Penitentiary (PFP). To this end, blood samples from human and cat participants will be tested for toxoplasmosis.
b) If you participate in the research, it will be necessary:
I - We hold a brief conversation with the application of a questionnaire regarding SARS-CoV-2 (which causes COVID-19), the presence of clinical alterations, the presence of cats in the PFP, human-animal interaction and their personal hygiene habits. Which can take an average of 30 minutes.
II - We will collect biological material (oropharyngeal content and blood) from you;

c) To this end, nurses from the Municipal Health Department of Piraquara will collect blood, in the presence of the responsible researchers;

d) It is possible that you experience some discomfort when the swab accesses the oropharynx/nasopharynx and especially with the prick of the needle for blood collection. If you are unable to bear the discomfort, the procedure will be stopped immediately.

e) Some risks related to the study may occur, such as: possible discomfort due to the time required to complete the questionnaire and collect material, or even possible embarrassment due to the content of the questions. If you wish, the procedure will be interrupted immediately.

f) The direct benefits expected from this research are to reduce the risk factors of the prison population and employees for SARS-CoV-2 and toxoplasmosis.

g) The researchers Alexander Welker Biondo, Gabriel Luís Brucinski Pinto and Louise Bach Kmetiuk, responsible for this study, can be located respectively at Rua dos Trabalhadores, 1540, Juvevê, Curitiba-PR, Department of Veterinary Medicine, room meetings (2nd floor) and by calling 3350-5812; Biological Sciences Sector Department of Cellular Biology, Jardim das Américas - Curitiba – PR; Avenida Coronel Francisco Heráclito dos Santos, 210, for any questions you may have and to provide you with any information you may need, before, during or after the end of the study, from Monday to Friday from 8 am to 6 pm. Researchers may also be contacted via email address and telephone number.

Researcher	Telephone	Email
Alexander Welker Biondo	(41) 3350-5812	abiondo@ufpr.br
Gabriel Luís Brucinski Pinto	(41) 99112-8169	gluisbp81@gmail.com
Louise Nicolle Bach Kmetiuk	(41) 99994-2216	louisebachk@gmail.com
Juliano Ribeiro	(41) 99241-6203	jpercicotti@gmail.com

h) Your participation in this study is voluntary and if you no longer wish to take part in the research, you may withdraw at any time and request that this signed Free and Informed Consent Form be returned to you.

i) Information related to the study may be known by authorized persons: Prof. Alexander Welker Biondo and Gabriel Luís Brucinski Pinto. However, if any information is disclosed in a report or publication, this will be done in encrypted form, so that your identity is preserved and confidentiality is maintained.

j) The material obtained – content of the oropharynx/nasopharynx and information from the questionnaire – will be used solely for this research and will be destroyed/discarded at the end of the study, within 4 years.

k) The expenses necessary for carrying out the research – collection material and exams – are not your responsibility and you will not receive any cash value for your participation.

l) When the results are published, your name will not appear, but a code.

m) If you have questions about your rights as a research participant, you can also contact the Ethics Committee for Research on Human Beings (CEP/SD) of the Health Sciences Sector of the Federal University of Paraná, by phone 3360-7259. The Research Ethics Committee is an independent multi and transdisciplinary collegiate body that exists in institutions that carry out research involving human beings in Brazil and was created with the aim of protecting research participants, in their integrity and dignity, and ensuring that researches are developed within ethical standards (Resolution nº 466/12 National Health Council).

I,____ have read this Consent Form and understand the nature and purpose of the study in which I have agreed to participate. The explanation I received mentions the risks and benefits. I understand that I am free to discontinue my participation at any time without justifying my decision and without any prejudice to myself . I voluntarily agree to participate in this study.

Piraquara, ______ of ____________________ of ____________

X ____
Signature of Research Participant or Legal Guardian

X ____
Signature of the Researcher in Charge or who applied the TCLE

B. Inmates: Interviewer: _________________ Date: ____/____/_______ Sample identification: ________

**1. Full name:**	**2. Sector/Cell:**
**3. CPF:**	**4. Date of birth:**
**5. Gender:** ( ) Female ( ) Other:	**6. City of Origin:**
**7. Marital Status:** ( ) Single ( ) Married ( ) Widowed ( ) Stable Union ( ) Separated	
**9. Ethnic data:** ( ) White ( ) Black ( ) Brown ( ) Indian ( ) Other	

10. Education:

( ) No literacy	( ) Incomplete elementary school	( ) Complete high school
( ) Incomplete primary school	( ) Elementary complete	( ) Incomplete higher
( ) Complete primary school	( ) Incomplete high school	( ) Graduated

11. Use of chemical agents: ( ) None ( ) Alcohol ( ) Tobacco ( ) Marijuana ( ) Cocaine ( ) Crack ( ) Synthetics

**12. Number of people in the cell:**	**13. Hours spent in cell:**
**14. Works in the penitentiary:** ( ) No ( ) Yes. **Function:**	**15. Weekly frequency:**

16. Contact with animals before detention: ( ) Yes. Species: ( ) No

17. Do you have contact with the cats in the penitentiary? ( ) Yes. Weekly frequency: ( ) No

18. Weekly frequency of going to the solarium:

19. Do you wash your hands before meals? ( ) Yes. How? ( ) No

20. Do you wash fruits and vegetables before consumption? ( ) Yes. How? ( ) No

**21. Do you eat raw or rare meat?** ( ) Yes. ( ) No. Species:	**22. Drinking water origin:**
**23. Are you pregnant?** ( ) Yes. Weeks: ( ) No	**24. Have you had children?** ( ) Yes, how many? ( ) No

25. Did you attend the penitentiary day care? ( ) Yes. **Time in months:** ( ) No

26. Have you ever had a miscarriage? ( )Yes Times: No ( ). **Cause?**

**27.** Does it have contact with earth or sand? ( ) Yes ( ) No	**28.** Habit of biting nails? ( ) Yes ( ) No
**29. Do you feel pain in your eyes?** ( ) Yes No	**30. Any other vision problems?** ( )No.( )Yes. **Which?**

31. Fever for more than 2 weeks without clinical diagnosis? ( ) Yes: ( ) Continuous ( ) Recurrent ( ) No

32. Have you ever been bitten by muquirana (body lice)? ( ) No. ( ) Yes. If so, in the penitentiary? ( ) Yes No

33. Bathing frequency: ( ) daily ( ) twice a week ( ) once a week ( ) once a month ( ) not informed

34. Frequency of changing clothes: ( ) ≥ 4 times a month ( ) 2-3 times a month ( ) 1 time a month

**35. Do you see rats in the penitentiary?** ( ) No ( ) Yes. **Local:**	**36. Ever been bitten by rats?** ( ) Yes No

37. Have you had contact with someone suspected/positive for COVID -19? ( ) Yes. **When?**
( ) No

**38. Do you use a face mask?** ( ) Yes No	**39. Temperature:**

40. Have you had any of the clinical signs below in the last 3 months?

( ) Fever	( ) Headache	( ) Conjunctivitis
( ) Dry cough	( ) Diarrhea	( ) Breathing difficulty
( ) Fatigue	( ) Abdominal pain	( ) Loss of smell/taste
( ) Chest pain	( ) Body/joint pain	( ) Loss of speech or movement

**Symptoms onset date:**	**Do you still have any other symptoms?** ( ) Yes. Which? ( ) No

Fill with system data:

Have you been tested for COVID-19 (SARS-CoV-2)? ( ) Yes. Date? ( ) No

If yes, which test? ( ) Quick test (blood). **Result:** ( ) PCR (swab). Lacen ( ) UFPR ( ) . **Result: CT:**

Regimen type/time:

Comorbidities :

Comments:

TERMS OF FREE AND INFORMED CONSENT - Inmates

We Prof. Alexander Welker Biondo (researcher in charge), Gabriel Luís Brucinski Pinto (Doctoral student) from the Federal University of Paraná responsible for the research, as well as project participant Louise Bach Kmetiuk (Doctor), Juliano Ribeiro (Doctor), we are inviting you to participate in the studies entitled "Prevalence of anti- *Toxoplasma gondii* antibodies in a prison population and potential risk factors" and "Molecular and serological monitoring of SARS- CoV -2 in female prisoners, prison officers and contact cats of the Paraná Women's Penitentiary".

a) The objective of this research is to evaluate the exposure of inmates, penitentiary agents and cats in contact with the Female Penitentiary of Paraná to SARS- CoV -2 through molecular detection. For this purpose, samples will be collected with swabs from the oropharynx/nasopharynx of detainees, employees and cats to verify the presence of the infectious agent. As well as to evaluate the prevalence of anti-T *oxoplasma gondii* antibodies in the occupants of the Piraquara Women's Penitentiary (PFP). To this end, blood samples from human and cat participants will be tested for toxoplasmosis.

b) If you participate in the research, it will be necessary:
I - We hold a brief conversation with the application of a questionnaire regarding SARS- CoV -2 (which causes COVID -19), the presence of clinical alterations, the presence of cats in the PFP, human-animal interaction and their personal hygiene habits . Which can take an average of 30 minutes.
II - We will collect biological material (oropharyngeal content and blood) from you;

c) To this end, nurses from the Municipal Health Department of Piraquara will collect blood, in the presence of the responsible researchers;

d) It is possible that you experience some discomfort when the swab accesses the oropharynx/nasopharynx and especially with the prick of the needle for blood collection. If you are unable to bear the discomfort, the procedure will be stopped immediately.

e) Some risks related to the study may occur, such as: possible discomfort due to the time required to complete the questionnaire and collect material, or even possible embarrassment due to the content of the questions. If you wish, the procedure will be interrupted immediately.

f) The direct benefits expected from this research are to reduce the risk factors of the prison population and employees for SARS- CoV -2 and toxoplasmosis.

g) The researchers Alexander Welker Biondo, Gabriel Luís Brucinski Pinto and Louise Bach Kmetiuk , responsible for this study, can be located respectively at Rua dos Trabalhadores, 1540, Juvevê , Curitiba-PR, Department of Veterinary Medicine, room meetings (2nd floor) and by calling 3350-5812; Biological Sciences Sector Department of Cellular Biology, Jardim das Américas - Curitiba – PR; Avenida Coronel Francisco Heráclito dos Santos, 210, for any questions you may have and to provide you with any information you may need, before, during or after the end of the study, from Monday to Friday from 8 am to 6 pm. Researchers may also be contacted via e- mail address and telephone number:

Researcher	Telephone	Email
Alexander Welker Biondo	(41) 3350-5812	abiondo@ufpr.br
Gabriel Luís Brucinski Pinto	(41) 99112-8169	gluisbp81@gmail.com
Louise Nicolle Bach Kmetiuk	(41) 99994-2216	louisebachk@gmail.com
Juliano Ribeiro	(41) 99241-6203	jpercicotti@gmail.com

h) Your participation in this study is voluntary and if you no longer wish to take part in the research, you may withdraw at any time and request that this signed Free and Informed Consent Form be returned to you.

i) Information related to the study may be known by authorized persons: Prof. Alexander Welker Biondo and Gabriel Luís Brucinski Pinto. However, if any information is disclosed in a report or publication, this will be done in encrypted form, so that your identity is preserved and confidentiality is maintained.

j) The material obtained – content of the oropharynx/nasopharynx and information from the questionnaire – will be used solely for this research and will be destroyed/discarded at the end of the study, within 4 years.

k) The necessary expenses for carrying out the research – collection material and exams – are not your responsibility and you will not receive any cash value for your participation.

l) When the results are published, your name will not appear, but a code.

m) If you have questions about your rights as a research participant, you can also contact the Ethics Committee for Research on Human Beings (CEP/SD) of the Health Sciences Sector of the Federal University of Paraná, by phone 3360-7259 or speak to responsible for the cell, for subsequent contact with the competent body. The Research Ethics Committee is an independent multi and transdisciplinary collegiate body that exists in institutions that carry out research involving human beings in Brazil and was created with the aim of protecting research participants, in their integrity and dignity, and ensuring that researches are developed within ethical standards (Resolution nº 466/12 National Health Council).

I,_ ____ have read this Consent Form and understand the nature and purpose of the study in which I agreed to participate. The explanation I received mentions the risks and benefits. I understand that I am free to discontinue my participation at any time without justifying my decision and without any prejudice to myself . I voluntarily agree to participate in this study.

Piraquara, ______ of ____________________ of ____________

X ______________________________________
Signature of Research Participant or Legal Guardian

X ______________________________________
Signature of the Researcher in Charge or who applied the TCLE
